# Renal compression caused by a giant retroperitoneal appendiceal abscess in a child: a rare case report and literature review

**DOI:** 10.3389/fped.2025.1630370

**Published:** 2025-09-17

**Authors:** Yuan-fei He, Shi-qin Qi, Jian Bian, Cheng-xiao Zhou, Yong-peng Duan

**Affiliations:** Department of Pediatric Surgery, Anhui Provincal Children’s Hospital, He Fei, Anhui, China

**Keywords:** appendiceal abscess, retroperitoneal, kidney, pediatric, drainage

## Abstract

**Objective:**

To enhance understanding of the diagnosis and management of retroperitoneal appendiceal abscesses in children.

**Methods:**

A retrospective analysis was conducted on the clinical data and surgical approach of a pediatric patient with renal compression caused by a giant retroperitoneal appendiceal abscess, treated in the Department of Pediatric Surgery at our Hospital. A review of the relevant literature was also performed.

**Results:**

After confirming the diagnosis of a retroperitoneal appendiceal abscess through comprehensive abdominal examinations, the patient successfully underwent retroperitoneal percutaneous catheter drainage under ultrasound guidance. The procedure lasted 25 min, with approximately 300 ml of pus drained intraoperatively. Post-drainage, the patient's fever and abdominal pain significantly improved. The patient was discharged in good condition after a 12-day postoperative hospital stay, with a recommendation to undergo appendectomy 3 months later.

**Conclusion:**

This case provides valuable insight into the diagnosis and surgical management of retroperitoneal appendiceal abscesses in children. For patients presenting with retroperitoneal abscesses, it is crucial to actively investigate appendiceal origin as a potential cause.

## 1 Introduction

Appendicitis is one of the most common causes of acute abdomen in children and can occur at any age. It accounts for approximately 20%–30% of pediatric hospital admissions due to acute abdominal pain (1). The etiology of appendicitis includes obstruction by appendicoliths, bacterial translocation, and anatomical factors (2). In children, the younger the age, the more rapidly appendicitis tends to progress, often leading to perforation and subsequent formation of an appendiceal abscess (3). Retroperitoneal appendiceal abscesses are easily misdiagnosed as other conditions. Here, we report an extremely rare case of a giant retroperitoneal appendiceal abscess causing renal compression in a pediatric patient.

All procedures performed in studies involving human participants were in accordance with the ethical standards of the institutional and/or national research committee and with the 1964 Helsinki Declaration and its later amendments or comparable ethical standards. Written informed consent was obtained from the patient's legal guardian for publication of all presented materials.

## 2 Materials and methods

### 2.1 Clinical data

**Figure 1:**
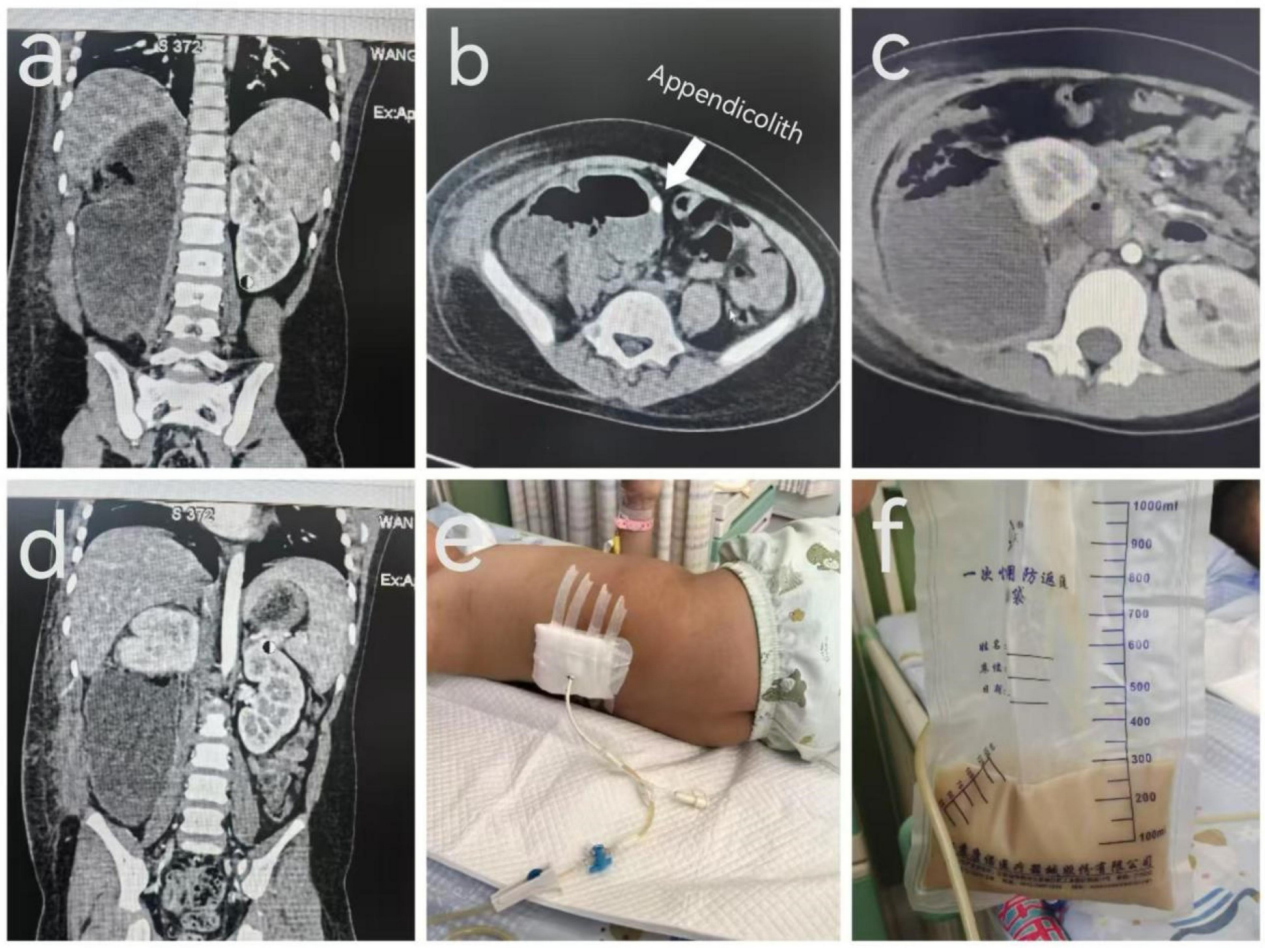
Preoperative CT nnnand intraoperative drainage images of a 5-year-old boy with a retroperitoneal appendiceal abscess. **[(a)** Preoperative CT image showing the maximal extent of the abscess. **(b)** Appendicolith. **(c)** Displaced kidney. **(d)** Compressed kidney. **(e)** Catheter drainage placement. **(f)** Drained purulent fluid].

A 5-year-old boy was admitted to our hospital at 19:08 on April 6, 2025, with the chief complaint of right-sided lumbar and abdominal pain accompanied by fever for six days.Six days prior to admission, the patient developed intermittent fever without a clear cause, accompanied by multiple episodes of vomiting of gastric contents. Abdominal pain subsequently appeared, predominantly in the right lower abdomen and right lumbar region. Urination was normal, with no hematuria or dysuria. Stool was yellow, loose, and of small volume. He received empirical anti-infective treatment at a local hospital for three days (diagnosis and medications unknown), with no significant improvement. He then presented to our outpatient clinic. Ultrasound indicated a retroperitoneal abscess, with a suspected appendiceal abscess. The patient was admitted with a preliminary diagnosis of “retroperitoneal abscess”. During the illness, the patient had no cough or nasal discharge, exhibited poor appetite, and appeared mildly lethargic. Physical examination: The patient was conscious and responsive, with a distressed facial expression. The abdomen was soft. There was fullness and deep tenderness in the right lower abdomen and right lumbar region. The psoas sign was positive. No palpable masses were detected, and digital rectal examination revealed no significant abnormalities. Auxiliary examinations: Emergency abdominal ultrasonography revealed significant bowel gas and poor acoustic transmission. The appendix was not clearly visualized. Two heterogeneous hypoechoic areas were seen in the right abdomen, measuring approximately 125 × 63 × 89 mm^3^ and 57 × 24 × 47 mm^3^, respectively, containing turbid fluid. The omentum was notably thickened with increased echogenicity. Multiple strip-like adhesions were observed in the surrounding bowel spaces, and bowel peristalsis was reduced. Findings were consistent with a retroperitoneal abscess, likely secondary to an appendiceal abscess. After admission, given the limitations of ultrasound, we performed a plain and contrast-enhanced abdominal CT to better delineate the abscess and plan the drainage approach. The child's renal function was normal before the scan (creatinine 21.6 µmol/L), and the CT was completed without sedation. The CT showed a large irregular mass was observed in the right abdominal cavity, containing extensive fluid-density and gas-density areas. The lesion extended from below the liver to the right iliac fossa, measuring approximately 160 × 85 × 56 mm^3^ (Figure 1a). A nodular high-density shadow was noted near the midline of the lesion's lower wall, with a non-contrast CT value of approximately 810 HU (Figure 1b). Contrast enhancement revealed marked enhancement of the cyst wall, obscured surrounding fat planes, increased mesenteric vascularity, and several enlarged lymph nodes at the mesenteric root. The right kidney was displaced medially and superiorly (Figure 1c), with compression and an arcuate indentation on the lower pole (Figure 1d); the renal pelvis appeared distended. The left kidney showed no significant abnormalities.Based on the clinical history and imaging findings, the diagnosis was confirmed as “retroperitoneal appendiceal abscess”.

### 2.2 Surgical procedure

Under local anesthesia, the patient underwent ultrasound-guided retroperitoneal percutaneous catheter drainage. Prior to the procedure, the patient's condition, the purpose of the intervention, and potential risks were fully explained to the patient's guardians. After obtaining informed consent, the procedure was performed in the dressing room under ultrasound guidance. Ultrasound localization of the right back revealed a heterogeneous hypoechoic area in the right retroperitoneal region measuring approximately 160 × 82 × 66 mm^3^, containing turbid fluid. Under ultrasound guidance, the puncture site was identified. After routine disinfection and draping, local infiltration anesthesia was administered with 1% lidocaine injection (trade name: Xylocaine). The puncture needle was inserted into the abscess cavity, and yellowish-white viscous pus was aspirated. A guidewire was introduced through the puncture needle. After removal of the needle, a 12F drainage catheter was inserted along the guidewire into the abscess cavity. Following withdrawal of the guidewire, yellowish-white purulent fluid was again aspirated through the catheter. Ultrasound confirmed the catheter was correctly positioned within the abscess cavity. The catheter was secured in place, hemostasis was achieved by compressing the puncture site, and sterile dressing was applied. The drainage catheter was connected to an external drainage system (Figure 1e). Throughout the procedure, the patient's vital signs remained stable, and no signs of discomfort were observed.

## 3 Results

The patient successfully underwent ultrasound-guided retroperitoneal percutaneous catheter drainage. The procedure lasted 25 min, and approximately 300 ml of purulent fluid was drained intraoperatively (Figure 1f). Following drainage, the patient's fever and abdominal pain significantly improved. Postoperative laboratory tests showed marked decreases in infection markers including white blood cells, neutrophils, and C-reactive protein (CRP). Platelet levels increased slightly. The platelet elevation was likely a rebound response following resolution of inflammation. During infection, inflammatory cytokines suppress hematopoietic stem cell activity in the bone marrow, affecting platelet production. Once the infection is controlled and inflammation subsides, hematopoiesis resumes, stimulating platelet generation and leading to increased platelet counts (Table 1). Although renal function was within the normal range before and after surgery, it showed improvement postoperatively (Table 2). Postoperatively, the patient received anti-infective and supportive treatment. A total of approximately 100 ml of pus was drained over 10 days via the catheter. The patient was discharged in improved condition after a 12-day hospital stay. At one-month follow-up, the patient showed no signs of discomfort, and no recurrence of the retroperitoneal abscess was observed.

**Table 1:** Pre- and postoperative changes in complete blood count (CBC).

Indicator	Preoperative	Postoperative day 3	Postoperative day 7
White Blood Cell (WBC)	20.74 × 10^9^ /L	17.76 × 10^9^ /L	10.53 × 10^9^ /L
Neutrophil(NEU)	15.13 × 10^9^ /L	14.17 × 10^9^ /L	6.24 × 10^9^ /L
Platelet(PLT)	451 × 10^9^ /L	570 × 10^9^ /L	692 × 10^9^ /L
Reactive Protein (CRP)	92.00 mg/L	69.45 mg/L	6.09 mg/L

**Table 2:** Pre- and postoperative changes in renal function.

Indicator	Preoperative	Postoperative day 3
Creatinine (Cr)	21.6 umol/L	12.1 umol/L
Blood Urea Nitrogen (BUN)	1.9 umol/L	1.9 umol/L
Uric Acid (UA)	216 umol/L	200 umol/L

## 4 Discussion

Retroperitoneal appendiceal abscess (RAA) is a rare complication of appendicitis. Due to its unique anatomical location, it is often misdiagnosed as a renal condition, such as a renal abscess, pyelonephritis, or even a retroperitoneal tumor (4). Retroperitoneal abscesses are clinically challenging to identify and diagnose. They may spread rapidly to the perirenal space, psoas muscle, abdomen, or even lower limbs, making them a potentially life-threatening complication (5). In this case, the child presented with right-sided lumbar and abdominal pain accompanied by fever, but without the classic migratory pain to the right lower quadrant. This reflects the insidious nature of retroperitoneal appendiceal abscesses. The absence of typical symptoms led to a delayed diagnosis and the formation of a large retroperitoneal abscess compressing the kidney. The retroperitoneal location of the appendix makes it less likely to present with typical signs of peritonitis. When inflammation irritates the psoas or obturator internus muscles, a positive psoas sign or obturator sign may be observed, rather than the classic abdominal tenderness or rebound pain (6). In this patient, a positive psoas sign, along with abdominal CT findings of retroperitoneal fluid collection, appendiceal perforation, and an appendicolith, provided critical diagnostic clues.

The ability of abdominal ultrasound to identify the source of retroperitoneal abscesses is affected by various factors, including abdominal fat and bowel gas. Computed tomography (CT) is therefore the preferred imaging modality, with a diagnostic accuracy for retroperitoneal abscess approaching 100% (5).In this case, abdominal non-contrast CT precisely revealed the location, extent, and anatomical relationships of the abscess. Enhanced CT showed indistinct fat planes, suggestive of infection rather than neoplasm. The renal pelvis appeared full, but no urinary calculi were identified. Multiplanar CT reconstruction traced the abscess back to the retroperitoneal appendix and identified an appendicolith. Differential diagnosis should include other retroperitoneal infections, such as perinephric or psoas abscesses. Laboratory tests including complete blood count, urinalysis, and CRP levels can assist in identifying the source of infection. In this patient, normal urinalysis helped exclude a urinary tract infection as the cause of the retroperitoneal abscess. Furthermore, abdominal CT provided critical guidance for selecting the surgical approach and drainage pathway. The primary treatment for retroperitoneal appendiceal abscess is surgical drainage. Attempting immediate appendectomy may risk injury to the ureter, intestines, or blood vessels, potentially resulting in serious complications. Therefore, initial drainage and infection control followed by interval appendectomy is considered a safer strategy (7). Conventional surgical approaches include open or laparoscopic drainage. In this case, we performed ultrasound-guided retroperitoneal catheter drainage under local anesthesia. This minimally invasive approach reduces surgical trauma and, with ultrasound guidance, helps avoid injury to blood vessels and intestinal structures. The drainage was performed via a retroperitoneal route rather than transabdominally, thereby minimizing the risk of peritoneal contamination and reducing the likelihood of postoperative adhesions. Pus culture and targeted antibiotic therapy postoperatively yielded satisfactory clinical results. The patient's fever and lumbar-abdominal pain significantly improved on the first postoperative day. One week after surgery, infection markers including white blood cell count, neutrophil percentage, and CRP normalized, and renal function improved, preventing further damage to the right kidney. Incomplete drainage of a retroperitoneal abscess may lead to complications such as septic shock, fistula formation, or chronic lower back pain. In this case, at one-month follow-up, re-examination confirmed that the abscess had fully resolved and the once-compressed right kidney had returned to normal position and shape, with the hydronephrosis disappearing and no new abnormalities, highlighting the importance of early diagnosis and precise drainage in improving prognosis. For patients in whom the appendix has not been removed, clinicians should remain vigilant for recurrence of appendicitis. Elective appendectomy is recommended once the infection is adequately controlled (8).

This case is extremely rare. A search of databases including PubMed and China's Wanfang Medical Database revealed that literature on retroperitoneal appendiceal abscesses is sparse. Du (9) reported a case in which a patient was initially hospitalized with upper respiratory tract infection and was later diagnosed with retroperitoneal appendiceal abscess after surgical consultation. The patient recovered after transabdominal drainage. Mustafa (10) reported a case of perforated retroperitoneal appendicitis complicated by Fournier's gangrene and retroperitoneal abscess. Azharuddin (11) presented a case of perinephric abscess secondary to appendicitis and noted that perinephric abscess caused by acute appendicitis is extremely rare, with few reports identifying the appendix as the source. Emad (5) described a case in which appendicitis led to retroperitoneal abscess, empyema, and Fournier's gangrene; the patient ultimately died due to irreversible septic shock and multiple organ failure.

Due to the rarity of reported cases, the exact timeline for retroperitoneal abscess formation secondary to appendicitis and the onset of clinical symptoms remains difficult to quantify. Although this is a single case report, it provides valuable clinical insight into the surgical management of retroperitoneal appendiceal abscess. It also offers an important differential diagnostic consideration for retroperitoneal abscesses of unclear origin.In patients presenting with abdominal pain, lumbosacral discomfort, and fever, but lacking typical abdominal signs, retroperitoneal appendiceal abscess should be considered. Early CT imaging is strongly recommended. For complex cases, multidisciplinary evaluation involving radiology, ultrasound, and surgery is crucial to determine the optimal surgical approach, reduce the risk of missed diagnosis, and prevent damage to critical structures. The surgical strategy should be tailored to the size of the abscess and the patient's overall condition, with infection control as the primary goal. Attempting one-stage appendectomy may increase surgical risk and should be avoided when not appropriate.

## Data Availability

The original contributions presented in the study are included in the article/Supplementary Material, further inquiries can be directed to the corresponding author.
